# Asbestos and disease – a public health success story?

**DOI:** 10.5271/sjweh.4146

**Published:** 2024-03-01

**Authors:** Bengt Järvholm, PhD, Alex Burdorf

**Affiliations:** 1 Department of Public Health and Clinical Medicine, Umeå University, Umeå, Sweden.; 2 Department of Public Health, Erasmus MC, Rotterdam, The Netherlands.

**Keywords:** exposure assessment, occupational health, occupational health prevention success, prevention, prevention strategy

## Abstract

**Objective:**

This paper discusses the failure and success of society to decrease the adverse health effects of asbestos exposure on workers’ health in relation to scientific knowledge.

**Methods:**

The findings are based on a narrative literature review.

**Results:**

Early warnings of the adverse health effects of workplace exposure to asbestos were published already in the 1930s. Serious health effects, such as malignancies and fibrosis due to occupational asbestos exposure, were highlighted in major medical journals and textbooks in late 1960s. New technologies could detect also asbestos fibers in the lung of non-occupational exposed persons in the 1970s. The first bans for using asbestos came in the early 1970s, and more general bans by authorities came in the 1980s and continue until today.

**Conclusions:**

The rather late recognition of adverse effects of asbestos exposure in the general population and measures to decrease the exposure through more general bans came rather late. However, the very strong measures such as general bans in many countries have been a success. A Swedish study showed that the general ban and other measures have decreased the risk of malignancies due to occupational exposure. The effect of the bans on adverse effects in the general population has yet to be studied. Analysis of fibers in the lungs of persons born after the bans could be an efficient method.

Asbestos has been used for thousands of years, eg, for increasing strength in clay pots (1). Asbestos is the commercial term for the mineral that is mined and processed for use in textiles, asbestos-cement tiles, insulation wool etc. A bizarre use was in the filter of cigarettes (2). The total consumption of asbestos was still around 1.3 million tons in 2022 (3). Russia was the major producer with 0.7 million tons.

The consumption of asbestos increased during the 20^th^ century (figure 1). It peaked at around 4.7 million tons in 1980 and has since then decreased (4). The time trends have varied depending on country and region. Between 1920 and 1950, more than half of the consumption was in North and Central America and the rest in Europe. In 1950, about 40% was used in Europe. The percentage increased to 64% in 1990 and then decreased to 35% in 2000, and thereafter dropped quickly in countries with a ban. This trend was countered by increasing use in Asia and Eastern Europe, including Russia.

**Figure 1:**
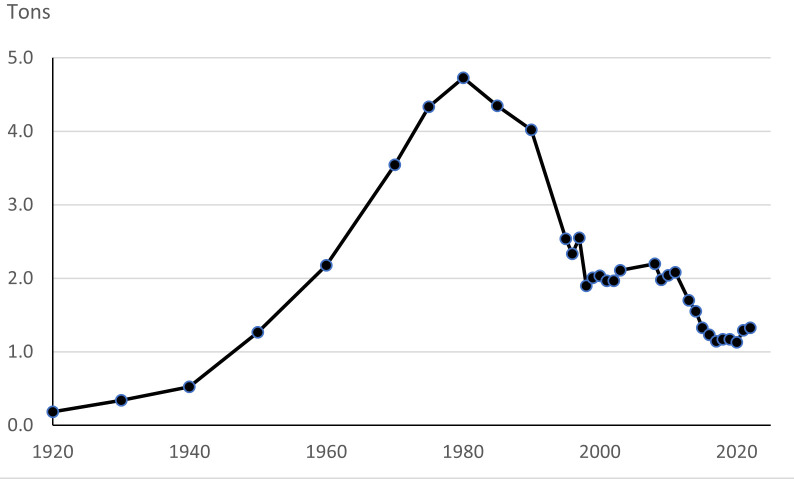
Estimated worldwide consumption of asbestos (from Vita R. Worldwide Asbestos Supply and Consumption from 1920 through 2022 (3, 4)

Asbestos is used for fireproofing, insulation, enforcing of cement and for products like brake linings. It can be woven into, for example, blankets and gloves due to its fibrous structure. It can be mixed with cement to produce plates and pipes or sprayed on steel for fireproofing. At its peak, asbestos was used in a large variety of products and, hence, is present in almost every workplace and many households.

Asbestos has been responsible for a substantial contribution to the general Global Burden of Disease (5) and is one of the most important contributors to the work-related Global Burden of Disease (6, 7). It has been estimated that around 255 000 deaths per year worldwide can be attributed to asbestos, thus, the mineral has a considerable impact on population health. Most of the deaths are attributed to occupational exposure (91%) and the rest through environmental exposure such as living close to an asbestos factory, having a family member that is occupationally exposed, or living in house with asbestos in the ventilation system.

Throughout history the scientific debate has been lively about the risks of asbestos, eg, whether it varies by type of asbestos, and if there is an interaction with other risk factors for disease in particular smoking. Analyses of lung tissue have mostly shown a higher concentration of amphibole fibers which has been interpreted as that they have a higher potential for causing cancer and especially mesothelioma (8, 9).

Due to its health effects, the use of asbestos has been restricted over the years, and today it is banned in many countries. This paper examines the success and failure of addressing the profound health effects of asbestos use in society. What were the driving forces for decreasing the risk of premature death and disease from asbestos and what were the arguments of those who have acted slower? We revisit the historical debates on the health risks of asbestos, the introduction of control measures, and ultimately the lessons we have learned how to cope with occupational health threats.

## Methods

This is a historical account on how science and society dealt with the adverse health effects of asbestos exposure. The literature search in PubMed used the terms “ban”, “restriction” and “asbestos”. Much of the literature about use and banning of asbestos could be found on websites, which were known to the authors, or found by reference lists.

### Adverse effects on health

*Persistence of inhaled fibers.*The adverse effects on health of asbestos are mainly through inhalation. Asbestos fibers are persistent to degradation and the fibers may stay for prolonged periods in the body. Asbestos fibers in the lung are often surrounded by proteins, which may contain iron and form “bodies” that are visible in light microscopy. Already in 1906, asbestos bodies were identified in the lungs, although at that time it was unknown that asbestos exposure was one of the causes of these bodies (10). Such bodies are called ferruginous bodies and may contain other types of fibers which cannot be separated from asbestos fibers with light microscopy. In more recent years, asbestos fibers have been identified in the lung with electron microscopy (11–13). The analytic procedures are technically challenging, and the concentration of fibers may vary within the lungs in an individual (14, 15). Measurements of fiber content in lungs during the 1980s and 1990s have found asbestos fibers in the general population. For example, around 20% of Finnish men, who were unlikely to have been occupational exposed to asbestos, had measurable concentrations of asbestos fibers in the lungs (16). Studies of fibers in asbestos bodies in non-occupationally exposed persons in Belgium showed that they contained asbestos fibers (17). Individuals in the general population of Vancouver with the highest levels (95% percentile) in the lungs, had around 40 million fibers each of chrysotile and tremolite in the lungs and around 400 000 fibers of amosite/crocidolite (12). Similar concentrations have also been found in non-occupational asbestos exposed persons in Texas (18). The occurrence of asbestos fibers in the lungs of the general population is an indication of the widespread use of asbestos. Its occurrence has been known for a long time and should be regarded as a marker for adverse health effects.

*Pleural plaques.* Pleural plaques are very common in older individuals with an occupational history of asbestos exposure. Pleural plaques are fibrous tissue mostly on parietal pleura and may sometimes be calcified. A Finnish study of construction and shipyard workers in 1990–92 reported that 80% of the workers had unilateral or bilateral pleural plaques, if they had been exposed to asbestos for at least 30 years (19). Similar findings were reported in Norway in the 1980s during a screening of the general population (20). The prevalence was around 25% among men where occupational asbestos exposure was uncertain, while it was around 60% in men with moderate or heavy exposure. The true occurrence of pleural plaques may be higher as the sensitivity for their detection by chest radiography is rather low. Especially for small plaques, sensitivity for detection is only between 13–46% compared to findings at autopsy (21–23). The occurrence increases by time since first exposure and dose (24, 25).

Pleura plaques are by far the most common pleural disorder caused by asbestos and are mostly localized in the parietal pleura. Other pleural disorders, such as effusions and pleuritis or fibrosis mostly affect the visceral pleura. The disorders may cause disability due to restrictive pulmonary function (26).

There has been a debate about the association between pleural plaques, malignancies, or asbestosis (27). Asbestos exposure is the common denominator, but it is still unclear if individuals with pleural plaques have an increased risk of other asbestos-related diseases compared to individuals with similar exposure but no pleural plaques.

*Asbestosis.* Exposure to asbestos may cause a chronic inflammatory process in the lungs with increasing fibrosis. The disability may vary, but most individuals with a severe form of this disease have a considerably shortened life expectancy (26, 28). Such cases almost exclusively occur among persons with occupational exposure to asbestos, hence the name of the disease. Cases of severe fibrosis were described in early 1900s and companies in the US and Canda had been requested (and declined) to cover insurance for workers exposed to asbestos already in 1918 (29). Asbestosis became an established disease in Britain in the 1930s with compensation arrangements (29, 30).

*Malignancies.* Several malignancies have been attributed to asbestos, but the strongest focus has been on mesothelioma and lung cancer. There are several case reports of associations between lung cancer, mesothelioma, and asbestos since 1930s (29, 31). The specificity of diagnosing mesothelioma has improved with modern immunological and molecular markers (32, 33). The diagnosis can be challenging, which in earlier periods may have hampered the recognition of its association with asbestos exposure, although the high attributable risk requires rather small studies to detect a significant association in case reference studies.

Solid evidence about the association between asbestos exposure and lung cancer originated from the studies by Doll in the 1950s and Selikoff and coworkers in the 1960s (34, 35). Case studies of mesothelioma in an area with asbestos mining in South Africa and cohort studies of insulators in the US in the 1960s established that asbestos was a major cause of mesothelioma (35–37). Studies of lung cancer among insulators found that the relative risk was similar among smokers and non-smokers, meaning that the absolute risk was much higher in smokers (38). However, the relative and absolute risks of mesothelioma linked with asbestos exposure was similar in smokers and non-smokers, a finding that has been supported by other studies (26, 28, 39). There has been an intense debate about differences in risk for malignancies with different types of asbestos with some arguing that the risk with chrysotile was low or absent (40). However, all types of asbestos were classified as carcinogenic to humans by IARC in 1987 and WTO reached the same conclusion in 2001 (39, 41).

It is estimated that more than 90% of all mesothelioma cases are attributable to occupational asbestos exposure, making them unique for follow-up on the adverse effects of asbestos exposure in the society (42, 43).

### Preventive measures

*Control measures.* The first control measures were introduced in the 1931 Asbestos Industry Regulations in the UK, but these regulations were not enacted across the total workforce; rather they focused on specific asbestos manufacturers. Protecting workers from asbestos exposure was the focus of control measures in the 1950s up to early 1980s. It included training of workers, standards for allowable concentrations at the workplace, prohibition to use dusty methods, and prohibition to use certain types of asbestos, such as crocidolite (44).

The early preventive measures intended to protect workers from high exposures were similar to those used for prevention of silicosis (45). The first standard of 2 fibers per cm^3^ in air was adopted in the 1969 Asbestos Regulations in Great Britain and was based on the risk of asbestosis. The estimated cumulative exposure that would cause that 1% of the exposed got fibrosis was estimated at 100 fiber years per cm^3^, and the standards were correspondingly adjusted, ie, to 2 fibers per cm^3^ for 50 years (46). The 2 f/ml limit in UK was adopted in several countries, eg, the first Swedish standard was from 1974. Already at the time of the 2 fibers per cm^3^ limit, there was a debate that it would only protect against asbestosis and not malignancies. A review in 1970 concluded it was “urgently necessary” to apply and enforce safer exposure limits based on information that was already available (47). The Swedish limit was lowered to 1 fiber per cm^3^ in 1978. However, the exposure–response curves were difficult to establish. There seemed to be a considerable difference between exposure–response for lung cancer caused by chrysotile between miners and textile workers (48, 49).

The European Union has recently decided that the standard should be 0.01 fibers per cm^3^ in its member states (50). It presumes that the states include rules that the measurements should include thin fibers. If not, the standard in the member states should be 0.002 fibers per cm^3^. Employers should also take steps to identify asbestos before maintenance work or demolition.

Measuring air concentration of fibers is challenging as there may be also other fibers, eg, from clothing. Previously, phase contrast microscopy was the standard method. To differentiate between types of fibers and measure very thin fibers requires electron microscopy. However, it is an advanced technique and findings may differ between laboratories (51). Application and removal of asbestos are usually done on temporary worksites, eg, construction of buildings, meaning that the exposure may vary strongly over days. We are not aware of any studies that have thoroughly investigated the variability of exposure between days, eg, in asbestos removals.

Early preventive measures also included health controls as chest radiographs to find early signs of fibrosis in line with prevention strategies among workers exposed to silica. The effects of the health examinations could mean that individuals were removed from exposure to asbestos in line with the praxis for health examinations of workers exposed to quartz. However, the removal could include not only persons with lung fibrosis, but also persons with pleural plaques (52). Many older workers with asbestos exposure have pleural plaques, and it is unclear if the pleural plaques per se means that the individual has an increased risk of asbestosis or cancer compared to workers without pleural plaques but with similar exposure to asbestos.

There has been an intensive debate in science and society about the association between different types of asbestos and malignancies. Some have emphasized that the risk with chrysotile is low and that it can be used with tolerable health risks (53). The first restrictions were to ban its use in especially dusty measures, such as spraying in shipyards by insulation workers. From the mid-1970s onwards evidence became stronger that carcinogenic potency differed across type of asbestos and cancer type. As a response, crocidolite was banned earlier in some countries.

The use of asbestos has varied over time and between countries, not only due to regulations and banning, but also due to industrial usage in particular industries. Thus, the potential impact on public health could vary between countries and can be regarded as a success in some countries and a failure in other countries. Although the differences in control measures between countries over time are well-described (54), less is known about the efficiency, ie, the effects on the health of the populations.

*Bans.*The use of asbestos can be banned mostly through regulation by governmental authorities. However, its use can also be banned by agreements between social partners (eg, employer organizations and unions). Management in a Swedish shipyard stopped using asbestos in shipbuilding in the early 1970s after they had recognized the health risks of asbestos exposure during a visit to the United States (personal communication Åke Sandén). Later, negotiations between Swedish unions and employers in the construction industry resulted in the end of asbestos use in new construction projects in the mid 1970s (55).

Spraying of asbestos can lead to high exposures both to the sprayer but also to other workers in the same area. Both Sweden and the US banned spraying of asbestos in 1973 (56). Denmark banned all use of asbestos except for roofing with asbestos-cement plates in 1980, but had already banned asbestos use in 1972 for insulation and waterproofing. Sweden, Iceland, and Norway banned most use of asbestos in the arly 1980s and then several countries have followed. The first banning usually included some exemptions, which later were withdrawn. Some bans included all types of asbestos, while some excluded chrysotile. In 2001, Canada entered into a legal dispute with the World Trade Organization to argue that chrysotile should be excluded from the ban in the European Union (41). Canada, which has been a major producer of asbestos, mainly chrysotile, banned all types at the end of 2018.

The use of asbestos has also decreased in countries that have not enacted a total ban. The US consumption of asbestos peaked in 1973 with around 800 000 tons, decreased to 217 000 tons in 1983, 32 000 tons in 1993, 4600 tons in 2003, 772 tons in 2013 and 290 tons in 2022 (3, 4). It was estimated that exposure to asbestos caused 12 000–15 000 per year in the US (57).

### Controversies

Controversies about preventive measures pertained to how fast they should be implemented, if they should include total banning, restrictions of certain types of asbestos, and acceptable exposure limits. Much of these controversies have involved scientists. The companies producing asbestos products have argued against bans, especially bans that included chrysotile (41). An important scientific debate focused on the magnitude of exposure–response relationships and subsequent derivation of threshold limit values. Exposure–response relationships have been difficult to establish since in many occupational cohorts few measurements in earlier periods were available and measurement technologies have been insufficient to measure thin fibers. Exposure–response relationships between miners and asbestos textile workers were very different, making it hard to determine occupational standards (8, 49). A systematic review with meta-analysis provided evidence that studies with higher-quality asbestos exposure assessment yielded higher meta-estimates of the lung cancer risk per unit of exposure. This review also indicated that potency differences for predominantly chrysotile versus amphibole asbestos-exposed cohorts were much smaller when meta-analyses were restricted to studies with the better exposure assessment strategies (58).

A heated scientific discussion focused on whether mesothelioma was only caused by amphiboles and not chrysotile. The lower persistence of chrysotile in the lungs has been used to argue against its importance for mesothelioma. Some researchers argued that the occurrence of mesothelioma in chrysotile miners was due to tremolite, an amphibole that occur together with chrysotile in the ore (59). Results from countries where mostly chrysotile had been used were questioned to be influenced by exposure to amphiboles, ie, introducing doubt (60). The “support” for chrysotile could be expressed as “Were it technically feasible to produce chrysolite free from fibrous tremolite a much safer product would probably result, at least so far as mesothelioma is concerned.” (59).

There are also controversies about compensation to individuals who have been exposed to asbestos, especially where they developed cancer. Typically, the controversy is about the size of exposure and how it can be evaluated (61–64). The companies may also suppress information about asbestos exposure (63). Although there may be some scientific consensus about the risk at some exposure (28), the final decision on compensation is done via judicial procedures.

### Successes and failures

It is obvious that research knowledge about the health risks of asbestos exposure was available from the late 1960s and early 1970s (47). Measures to prevent health effects were similar to the prevention of silicosis, ie, focused on heavy-exposed workers. There were also studies showing that exposure to asbestos was widespread in the general population and that low exposure may have importance for the occurrence of lung cancer (65). Such widespread exposure, biopersistence of the fibers and the well-established risk of malignancies would today probably have led to banning or restrictions much faster than was the case for asbestos. Compared to measures and arguments taken against exposure to polychlorinated biphenyls (PCB), dioxins and perfluoroalkylated substances (PFAS) the measures against asbestos were much slower.

Major measures to decrease occupational exposure and exposure in the general population started to be enforced in the late 1970s and during the 1980s (44, 56, 57). The interaction between smoking and asbestos in the cause of lung cancer was established in the late 1960s and 1970s (66, 67). However, the importance of smoking was often emphasized before the risk of asbestos (27). For example, exposed workers were told to stop smoking before they were told about the risk of asbestos exposure. The delays of more general actions by authorities during the 1970s can be regarded as a failure. However, the actions taken later were rather strong and, in many countries, the measures can be regarded as a success.

There is a long latency between asbestos exposure and occurrence of mesothelioma. Thus, measuring the benefits of preventive measures on the occurrence of mesothelioma cannot be evaluated until several decades later. An analysis 30 years after the Swedish asbestos ban showed that men who started to work after the ban have a lower risk of pleural mesothelioma (68). Thus, the ban and other preventive measures are a success. This analysis is possible as asbestos is a major cause of pleural mesothelioma. It will be much harder to show and interpret a decreasing incidence of lung cancer, where there are other competing risk factors, such as smoking and radon exposure.

There is still asbestos exposure in countries with a complete ban, eg, during renovation or demolition of buildings with a high level of asbestos materials. Both workers and other individuals in the buildings may be exposed. Whether the present preventive measures are sufficient to protect the individuals will be hard to detect by studying the occurrence of mesothelioma as the cases will be few and occur many years after the exposure. Measuring the concentration of fibers in the air will also be challenging as the concentrations should be very low. Furthermore, measurements are hampered as asbestos removal work has short durations on temporary worksites. Studying exposure by the occurrence of asbestos fibers in the lungs during autopsy or surgery may be a more sensitive method. If the exposure to asbestos is almost eliminated, it is expected that the concentrations of asbestos fibers in the lungs of younger individuals in the general population and workers would be almost nil. However, we have not found any such studies. Thus, it will be a challenge to prove that the preventive measures so far, and in the future, have been a success.

### Concluding remarks

The preventive measures to decrease the risk of asbestos-related diseases is a success in many western countries today. The pace, especially during the period 1960–1990, could have been faster. However, the large use of asbestos in some other countries should be considered a failure.
